# The SARS-CoV-2/Receptor Axis in Heart and Blood Vessels: A Crisp Update on COVID-19 Disease with Cardiovascular Complications

**DOI:** 10.3390/v13071346

**Published:** 2021-07-12

**Authors:** Priya Veluswamy, Max Wacker, Dimitrios Stavridis, Thomas Reichel, Hendrik Schmidt, Maximilian Scherner, Jens Wippermann, Guido Michels

**Affiliations:** 1 Heart Surgery Research, Department of Cardiothoracic Surgery, Faculty of Medicine, Otto-von-Guericke University, 39120 Magdeburg, Germany; max.wacker@med.ovgu.de (M.W.); Dimitrios.Stavridis@st.ovgu.de (D.S.); Maximilian.Scherner@med.ovgu.de (M.S.); Jens.Wippermann@med.ovgu.de (J.W.); 2 Department of Cardiology, Diabetology and Infectiology, Klinikum Magdeburg, 39130 Magdeburg, Germany; Thomas.Reichel@Klinikum-Magdeburg.de (T.R.); Hendrik.Schmidt@Klinikum-Magdeburg.de (H.S.); 3 Department of Acute and Emergency Care, Sankt Antonius-Hospital Eschweiler, 52249 Eschweiler, Germany; Michels@SAH-ESCHWEILER.DE

**Keywords:** SARS-CoV-2, COVID-19, heart, blood vessels, thrombosis, glycocalyx, bradykinin, cardiovascular disease

## Abstract

The SARS-CoV-2 virus causing COVID-19 disease has emerged expeditiously in the world and has been declared pandemic since March 2020, by World Health Organization (WHO). The destructive effects of SARS-CoV-2 infection are increased among the patients with pre-existing chronic conditions and, in particular, this review focuses on patients with underlying cardiovascular complications. The expression pattern and potential functions of SARS-CoV-2 binding receptors and the attributes of SARS-CoV-2 virus tropism in a physio-pathological state of heart and blood vessel are precisely described. Of note, the atheroprotective role of ACE2 receptors is reviewed. A detailed description of the possible detrimental role of SARS-CoV-2 infection in terms of vascular leakage, including endothelial glycocalyx dysfunction and bradykinin 1 receptor stimulation is concisely stated. Furthermore, the potential molecular mechanisms underlying SARS-CoV-2 induced clot formation in association with host defense components, including activation of FXIIa, complements and platelets, endothelial dysfunction, immune cell responses with cytokine-mediated action are well elaborated. Moreover, a brief clinical update on patient with COVID-19 disease with underlying cardiovascular complications and those who had new onset of cardiovascular complications post-COVID-19 disease was also discussed. Taken together, this review provides an overview of the mechanistic aspects of SARS-CoV-2 induced devastating effects, in vital organs such as the heart and vessels.

## 1. Introduction

Rapidly evolving coronavirus (COVID-19) disease that has drenched millions of lives on our planet is caused by a novel strain of severe acute respiratory syndrome (SARS) coronavirus, which was first reported in Wuhan city of Hubei Province in China, in late 2019 [1]. The World Health Organization (WHO) has declared thoughtful alertness of the global pandemic during a novel coronavirus outbreak on 11 March 2020 [2]. The de novo sequences of 2019-novel (n) CoV genome obtained from lung lavages of infected patients has revealed a distinct clade that are clustered together within the sarbecovirus subgenus with a novel lineage β (beta) coronavirus organization and is reported similar to two types of bat-derived SARS such as coronavirus, ZC45 and ZXC21 [3], exhibiting a rapid contagiousness with an accelerated human-to-human transmission [4]. Despite several viral antigenic components, the spike (S) proteins present on the surface of the SARS-CoV-2 are majorly responsible for corona associated pathogenesis, by firmly attaching to the receptors on the host cell membrane [5,6]. Until today, angiotensin converting enzymes-2 (ACE2) and transmembrane protease, serine 2 (TMPRSS2) serve as two major SARS-CoV-2 functional host cellular entry receptors [7]. In addition to TMPRSS2, cathepsin L (CTSL), furin contributes in assisting the cleavage of S protein with facilitating the virus cellular entry [8,9]. Very recently, the expression of tyrosine-protein kinase receptor UFO (AXL) receptor on host pulmonary and bronchial epithelial cells has been shown to promote SARS-CoV-2 infection [10]. The S protein of SARS-CoV-2 binds to the human ACE2 receptor with a dissociation constant (KD) of 14.7 nM in comparison to the S protein of SARS-CoV, which is 325.8 nM [11], indicating a higher sensitivity of SARS-CoV-2 (S) protein binding affinity to ACE2 than SARS-CoV. The availability and heterogeneous expression of these cognate receptors that bind to the SARS-CoV-2 virus provoke infectivity and transmissibility of the virion to afflict multiple organs [12,13]. Furthermore, the expression of these receptors varies between individuals and between different organs [13]. The COVID-19 disease manifests from self-limiting, mild to extremely severe with accelerated mortality, where underlying comorbid conditions remain to be the decisive factor in disease progression and outcome [14,15]. This review majorly focuses on the involvement of SARS-CoV-2/receptor axis in the pathophysiological outcome of vital organs, heart and blood vessels. This includes SARS-CoV-2 receptor expression pattern and function, SARS-CoV-2 tropism and cellular residence and a potential SARS-CoV-2 induced genesis of vasculopathy associating leakage and thrombosis. This review also provides a cumulative overview of evidence based clinical data obtained from patients with COVID-19 disease, who (i) harbored pre-existing cardiovascular diseases and (ii) developed cardiac-related complications, during the pandemic. 

## 2. SARS-CoV-2 and Its Receptors in Cardiac Pathophysiology 

### 2.1. SARS-CoV-2 Receptor Expression and Virion Tropism and Outcome in Heart Tissues

In terms of anatomy and physiology, the heart is one of the complex and key organs with four distinct functional cardiac chambers and has been decoded for heterogenous cell population content that includes ventricular cardiomyocytes, atrial cardiomyocytes, cardiac fibroblasts, endothelial cells (ECs), pericytes, smooth muscle cells (SMCs), neuronal cells (NCs), adipocytes, mesothelial cells, lymphoid and myeloid cells [16], where cardiomyocytes are the most abundant cell type. While the pattern of cellular expression of SARS-CoV-2 receptors, ACE-2 and TMPRSS2, were found to be differential and higher in the adult human heart than fetal heart, minimal expressions were noticed in the adult coronary arteries [17]. Interestingly, the adult heart exhibited the highest percentage of ACE-2 positive SMCs (10.38%) that is followed by the expression of ACE-2 in cardiac fibroblast (5.38%), cardiomyocytes (4.98%), ECs (2.96%) and macrophages (MΦ) (1.97%) [17]. Other studies have evidently reported the highest expression of ACE2 on cardiac pericytes that are crucial for myocardial microcirculation and since they are located on the exterior surface of capillaries or venules, unlike the SMCs that are aligned inside the arteries [18], pericytes are more susceptible to SARS-CoV-2 attack in the human heart [19]. However, the fetal heart exhibited the highest percentage of ACE-2 positive mast cells, followed by red blood cells (RBCs), cardiomyocytes, T and B cells, MΦ, cardiac fibroblast and ECs [17]. The cardiomyocytes associated with ACE2 receptors are majorly localized at the plasma membrane [20]. Furthermore, the expression of another vital SARS-CoV-2 receptor, TMPRSS2, that primarily cleaves the (S) proteins of SARS-CoV-2 virus in aiding the fusion with host cell membrane was also evident in adult endothelial cells followed by cardiomyocytes and to certain extent in SMCs and MΦ. These TMPRSS2 are predominantly found in the nucleus and cytoplasm of both normal and diseased hearts with cardiomyopathy [20]. Though minimal expression of TMPRSS2 was found in the heart in comparison to the lung tissues, underlining a lesser extent, of heart ECs, cardiomyocytes and SMCs to SARS-CoV-2 infections [21], it is undoubtedly difficult to affirm the degree of vulnerability since other two SARS-Cov-2 (S) protein priming proteases (CSTL and FURIN), which compensates for lowered heart TMPRSS2 expressions, were evidently reported in the adult heart [17]. 

While high-throughput RNA sequencing and proteomic profiling has revealed an elevated heart tissue expression of ACE2 receptor, interestingly, a conventional based western blot method on myocardial biopsies has revealed an elevated expression of an additional SARS-CoV-2 receptor, AXL, in patients with heart failure [22], which reinforces the mechanism for effortless SARS-CoV-2 invasion and aggressiveness in pre-existing conditions. During myocardial infarction, the infarct associated MHC II^high^ cardiac macrophages exhibited increased AXL expression. While cardiac macrophage with AXL sufficiency retained inflammatory phenotype that was assisted with copious amount of pro-inflammatory cytokine productions, such as interleukin (IL)-1β, IL-6 and tumor necrosis factor-alpha (TNF-α), the AXL deficient cardiac macrophages were polarized into reparative MHC^low^ phenotype that was biased into anti-inflammatory cytokines productions, such as IL-10 and transforming growth factor- βeta (TGF-β). Moreover, lipopolysaccharide (LPS)-stimulated AXL positive MΦ induced the NLRP3 inflammasome, which was evaluated by cleaved caspase 1 (p10) with concomitant production of IL-1β, while the impaired activation of AXL negative MΦ was noticed with decreased tumor necrosis factor-alpha (TNF-α) production with circumstantial increase in IL-10, indicating the role of AXL in TLR-4 augmentation and inflammasome activation. Of note, AXL sufficient MΦ promoted enhanced glycolysis to compensate for the energy demands needed for sustaining the pro-inflammatory state in response to infection induced inflammatory stimuli. This glycolytic reprogramming that occurs inside cardiac MΦ is dependent on hypoxia inducible factor-1 alpha (HIF-1α) [23]. Both HIF-1α signaling and IL-1β production are independently achieved through the phosphorylation of STAT-1 activation that is promoted by AXL [23]. It was noted that AXL expression was correlated with the SARS-CoV-2 virus titer in COVID-19 patients, indicating upregulated AXL expression during the infection [10] and indeed virus replication are further amplified in glucose-rich micro-environment, where virus-induced metabolic alterations occur at the mitochondrial level with reactive oxygen species (ROS) production. These SARS-CoV-2 induced maladaptation favors the stabilization of HIF-1α to support their replication efficiency and to sustain the hyper-inflammatory status through the IL-1β production [24]. Of note, the SARS-CoV-2 virus is known to induce STAT-1 dysregulation [25]. These evidence-based reports could, in part, support the SARS-CoV-2 associated cardiac pathogenicity with AXL receptor manipulation. Moreover, in heart sections, the tropism of the SARS-CoV-2 virus was confirmed within the cardiomyocytes in the left atrium [20], where both actively transcribed virus RNA and viral proteins found inside cardiomyocytes induced cellular alterations, including increased cardiomyocyte volumes with intracellular edema, together with disarranged, and plurifocally clumped myofibrils. However, the sarcolemma and the mitochondria of cardiomyocytes remained undamaged, which does not indicate cardiomyocyte death [26]. In contrast to this, another report has found SARS-CoV-2 induced foci of cardiomyocyte necrosis and the virus has shown tropism towards endocardium endothelial cells, cardiac associated macrophages, neutrophils, and fibroblasts [27]. 

The tropism exhibited by the SARS-CoV-2 virus towards cardiac tissues due to several aforementioned SARS-CoV-2 receptors on the cardiac system, especially on cardiomyocytes and pericytes, could culminate in cardiac injuries, where the virus directly invades into the myocardial cells by binding to the ACE2 receptor to cause myocardial injuries. In addition to this, the bystander effect plays a significant role in mediating cardiac injuries, which include (a) systemic hyper responsiveness due to cytokine storm; (b) cardiac tissue hypoxia due to respiratory failure; (c) coronary plaque destabilization, (d) induction of coronary thrombosis, which are elicited by high SARS-CoV-2 load, are also responsible for cardiac muscle dysfunction and injuries, causing acute or fulminant myocarditis [28,29]. Though direct cardiac injury mediated by SARS-CoV-2 virus is not completely understood, it was evident that this virus does replicate inside cardiomyocytes and are localized at the perinuclear region of the cytoplasm, causing viral-mediated cytopathic effects, including apoptosis which was reflected by the cleaved caspase-3 expression and cessation of beating in 72 h post-infection, as demonstrated by beat rate contractility analysis [30]. These data were further supported at the transcriptional level, where RNA sequencing was performed to demonstrate the upregulation of signature genes involved in apoptosis and oxidative stress [31]. The underlying molecular mechanism reports that the double stranded RNA (dsRNA), the viral replication intermediates, of the SARS-CoV-2 virus are being sensed by the cytosolic protein kinase R (PKR) of cardiomyocytes, where PKR undergoes autophosphorylation prior to the phosphorylation of eukaryotic initiation factor 2 (elF2α), leading to cell apoptosis due to arrest in further translation [32]. The next major intracellular event that occurs during the course of SARS-CoV-2 infection is the oxidative stress, which dictates an increased ratio of pro-oxidants to antioxidants in a living system and is considered to be pivotal in determining the cell fate. Here, the SARS-CoV-2 viral RNA are presumed to be sensed by TLR-7 further activates NADPH oxidase 2 (NOX2) to produce reactive oxygen species (ROS). During viral infections, e.g., SARS-CoV-2 infection, the NOX associated ROS production induces inflammation and tissue damage. Therefore, NOX2 activation were described to be one of the underlying mechanisms for myocardial injury, as evaluated by the increased cardiac troponin (cTn1) [33,34]. In general, a counter balance mechanism is being induced via nuclear factor E2-related factor 2 (Nrf2) pathway as antioxidant responses to circumvent the excessive ROS levels. Here, Nrf2 which is associated in the cytoplasm with kelch-like ECH-associated protein 1 (Keap1) is inclined to proteasomal degradation, especially when the ROS level is minimal. However, Nrf2 dissociates from cytoplasmic Keap1 to translocate into the nucleus and bind to the promoters of genes encoding for antioxidant enzymes such as superoxide dismutases and glutathione peroxidases [33]. Nevertheless, the ROS induced cell injuries tend to inhibit the Nrf2 pathway, as evidenced by the suppression of Nrf2 related antioxidant gene expression during SARS-CoV-2 infection [35,36]. Taken together, these multiple events mediated by SARS-CoV-2/ACE2 axis intensify cardiac injuries in COVID-19 disease.

### 2.2. Atheroprotective Role of ACE2 Receptor, Its Variants and Association with SARS-CoV-2 Susceptibility to Cardiac Risk

One of the SARS-CoV-2 receptors, ACE2, a homolog for ACE, is a key negative regulator of the renin–angiotensin system (RAS) [37] that plays a significant role in protecting the cardiac functions, especially in curtailing prothrombotic events [38]. There exists a dynamic equilibrium between ACE and ACE2 under the steady-state condition, where the ACE2 acts as a counter regulator of ACE by converting angiotensin II to vasodilatory molecules, Ang 1–7, which is >300 times effective than converting angiotensin I to Ang 1–9. These vasodilatory ang 1–7 molecules [39] aid in protecting the endothelial cell functions by (i) decreasing the protein levels of adhesion molecules, vascular cell adhesion molecules (VCAM) and E-selectin; and (ii) decreasing the production of chemokine and cytokine, monocyte chemoattractant protein-1 (MCP-1) and IL-6 [40] (iii) limiting monocyte recruitment; (iv) attenuating NADPHox induced reactive oxygen species (ROS) generation, partly mediated through the decreased expression of p22phox, a vital component of superoxide-generating vascular NADH/NADPH oxidase [41,42]; (v) increasing eNOS and NO production (eNOS/NO) pathways with a concomitant decrease in NADPH oxidase 2 and oxidase 4 (Nox2 and 4) (Nox/ROS) pathways [43]. The antithrombotic and anti-inflammatory effects exerted by Ang 1–7 is mediated by binding to the Mas receptor [44,45] and by eliciting the production of NO and prostacyclin, an anticoagulant. Of note, Mas knockout has shown shorter bleeding time and increased thrombi size [46]. In addition, when SARS-CoV-2 binds to ACE2 causing insufficient ACE2 expression, the counterbalanced expression of Ang II activates NOX to generate ROS which leads to tissue injury [36]. Overall, the ACE2 receptor deficit has been shown to develop atherosclerosis in LDL^-/-^ mice [47], dictating that these aforementioned and validated observations affirm the atheroprotective role of the ACE2 receptor, which is mediated through the ACE2/Ang 1–7/Mas axis [46]. 

Despite atheroprotective attributes of ACE2, the underlying pathophysiological conditions such as diabetes, chronic obstructive pulmonary diseases (COPD), malignancies [48] and cofounding factors including cigarette smoking [49], and age [50] tend to upregulate ACE2 receptors which adds further complexity to becoming highly detrimental when binding to the SARS-CoV-2 virus. However, an enzymatic cleavage of ectodomain ACE2 receptor occurs by the action of a disintegrin and metalloproteinase 17 (ADAM-17) also known as tumor necrosis factor-a converting enzyme (TACE), generating active and soluble (s) forms of ACE2 [51,52]. Since angiotensin II is elevated during the heart failure condition, the activity of ADAM-17 is internally promoted by angiotensin II that is reported in several cardiac diseases, as a feedback system to sustain the ratio of angiotensin II/ACE2 expression [39]. The cleaved and shed sACE2 circulates in the plasma of patients with heart failure [53], aortic stenosis [54,55] and coronary artery disease (CAD) [56] and positively correlates with worsening disease prognosis. Of note, plasma sACE2 has been accounted as a highly ranked and independent determinant for cardiovascular mortality compared to other established cardiovascular risk factors [57]. Nevertheless, it has been demonstrated that the human recombinant sACE2 tends to decrease angiotensin II and concomitantly increases Ang 1–7 [58,59,60]. Furthermore, the generated soluble variant of ACE2 serves as a decoy receptor and binds to the spike proteins of SARS-CoV-2 virion in order to limit the virus uptake by membrane-bound ACE2 receptors [61]. These issues further interrogate whether the circulating native sACE2 could exert a protection against angiotensin II mediated cardiac dysfunction or could it effectively bind to SARS-CoV-2 spike protein as the patients with comorbid conditions, including heart failure or CAD are more vulnerable to the SARS-CoV-2 mediated severity, despite elevated sACE2 levels in their circulation. This links to the further query on whether these native sACE2 is functionally impaired due to the amino acid changes (polymorphisms) or variants, generating weak intermolecular interaction with virus spike protein [62]. Indeed, these variants contribute to different affinities of ACE2 with the spike protein receptor-binding domain (RBD) region, promoting disparity in the rates of infectivity. This could explain the reason behind the incapability of fencing SARS-CoV-2 virus, by a high concentration of plasma circulating naïve sACE2 in patients with underlying heart failure. 

## 3. SARS-CoV-2, and Its Receptors in Blood Vessel Pathophysiology

### 3.1. Endothelium-Expressed Receptors and Vascular Leakage during SARS-CoV-2 Infection

**Figure 1:**
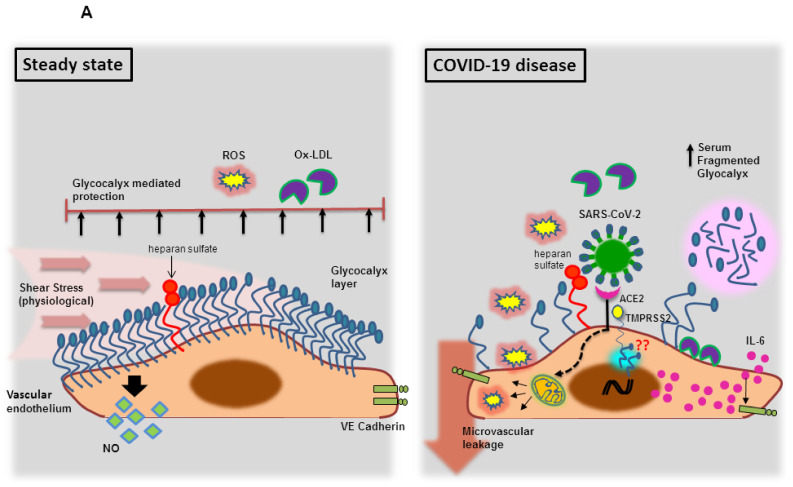

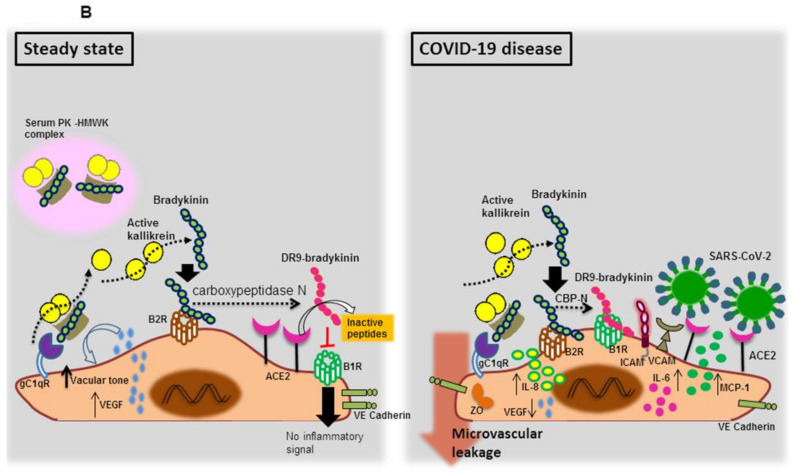
Potential molecular mechanisms underlying vascular leakage during SARS-CoV-2 infection. (**A**) Under the steady-state condition, vascular endothelium expresses qualitative and quantitative glycocalyx, which protects the endothelial damage with plasma-circulating ox-LDL and free oxygen radicals termed reactive oxygen species (ROS). In addition, these vascular endothelial cells tend to secrete nitric oxide (NO), which supports vasodilation and improved circulation, under physiological shear stress condition. In patients with the COVID-19 disease, there might be a heavy loss of the endothelial glycocalyx with possible impairment of glycocalyx re-synthesis. This is more evident when COVID-19 patients exhibited high levels of the fragmented glycocalyx. When a SARS-CoV-2 virus binds to angiotensin-converting enzyme 2 (ACE2) receptor expressed on endothelial cells, they might tend to induce mitochondrial ROS production, which decreases the junctional proteins, such as vascular endothelial (VE)-cadherin expression, and increases the production of IL-6 pro-inflammatory cytokines. Due to the loss of the glycocalyx layer, plasma-circulating ROS and ox-LDL can easily damage the endothelial cell membrane, causing endothelial dysfunction. Of note, the SARS-CoV-2 virus utilizes a component of the glycocalyx layer, one of the glycosaminoglycan side chains called heparan sulfate, as a cofactor to assist spike protein-mediated viral invasion. These SARS-CoV-2-induced changes in endothelial cells might lead to vascular leakage. For S protein priming the transmembrane protease, serine subtype 2 (TMPRSS2) is used. (**B**) Under the steady-state condition, the serum-circulating pre-kallikrein (PK) that are complexed with high molecular weight kininogen (HMWK) bind to endothelial-expressed receptors gC1qR, where PK activates into kallikrein that liberates bradykinin, an inflammatory component, from HMWK. These generated bradykinins are also processed to generate DR9-bradykinin by carboxypeptidase N (CBP-N). The bradykinin and the metabolite DR9-bradykinin bind to the bradykinin receptors B2R and B1R, respectively. However, ACE2 receptors bind to DR9-bradykinin and convert it into an inactive peptide to prevent inflammation. In patients with the COVID-19 disease, the SARS-CoV-2 virus hijacks ACE2 and is thereby competing with DR9-bradykinins. These ACE2 unbound DR9-bradykinins remain free to bind to B1R to induce a devastating inflammatory signal in the endothelial cells by (i) upregulating IL-6, MCP-1 and IL-8; (ii) upregulating intracellular adhesion molecule (ICAM) and vascular cell adhesion molecule (VCAM); (iii) downregulating vascular endothelial growth factor (VEGF), which is required for vascular tone; (iii) decreasing VE-Cadherin required for junctional integrity. Moreover, tight junctional proteins, such as zonula occludens (ZO), are also reduced. These endothelial dysfunctions might lead to microvascular leakage during COVID-19 disease.

Next to the expression pattern on cellular fractions of the human heart, ACE2 receptors were shown to be expressed in vessel associated endothelial cells from small and large arteries and veins in all the tissues [12]. Compared with adult and fetal heart, coronary arteries exhibited the lowest expression of ACE-2 receptors, including fibroblasts, SMCs, ECs and in pericytes of intramyocardial microvessels [17]. The TMPRSS2 protein was readily found in the endothelium of coronary arteries and in pericytes of intramyocardial microvessel [20]. Of note, human endothelial cells also upregulate an AXL receptor during viral infection [63]. However, the SARS-CoV-2 receptor expression varies among microvascular and macrovascular beds between different organs [64]. These endothelial expression of SARS-CoV-2 associated receptors increase the vulnerability of the inner layer of the vessel to virus attack and, indeed, the SARS-CoV-2 infected patient has been reported with severe microvascular blood vessel leakage [65,66], which acts as an exit window for SARS-CoV-2 viruses to invade other organs. Such microvascular leakage could be mechanistically linked to the (i) endothelial glycocalyx dysfunction [67] and (ii) bradykinin receptors (B1R) trigger via kallikrein-*kinin* system (KKS) activation [68], during the interaction between SARS-CoV-2 and its receptor(s). The schematic illustration of molecular mechanisms behind vascular leakage that possibly occurs during COVID disease is shown in Figure 1A,B. 

#### 3.1.1. Endothelial Glycocalyx Dysfunction during SARS-CoV-2 Infection

Vascular endothelial glycocalyces are the membrane-bound macromolecules that are attached to the endothelial cell membrane and are composed of core proteins, which consist of membrane-anchored proteoglycans (syndecan, glypican), long-chain hyaluronic acid (HA) and glycosaminoglycan side chains (heparan sulfate, chondroitin sulfate) [69]. These glycocalyces serve as “sentinels” against inflammation by preserving endothelial cell functions associated with (i) the vascular permeability barrier; (ii) blood flow homogeneity; (iii) the protection of ECs from oxidants, pro-inflammatory cytokines and inflammatory immune cells [70]. Further, mechanotransduction of shear stress stabilizes glycocalyx, which is crucial for physiological nitric oxide (NO) synthesis from endothelial (e) NOS [70]. An impaired endothelial glycocalyx is reported among patients with underlying comorbidities, including diabetes, obesity, chronic kidney disease, cardiovascular disease [71] as well as in people with advancing age [70] and smoking habits [71]. Intriguingly, SARS-CoV-2 patients exhibited elevated levels of fragmented vascular endothelial glycocalyces [72]. It has been proposed that shedding of glycocalyx occurs due to an evolutionary mechanism that can be newly synthesized from the golgi apparatus of the same cells for regulating endothelial homeostasis [73]. This is obviously evident when the SARS-CoV-2 virus uses one of the glycosaminoglycan side chains, heparan sulfate, as an assisting cofactor for the spike protein mediated viral entry [74], in addition to ACE2, expressed on endothelial cells [12]. While it is uncertain to expect re-synthesis of glycocalyx when it is being utilized by SARS-CoV-2 virus infection under pre-existing chronic conditions, the patchy glycocalyx layer are easily targeted by ROS and cytokines, especially IL-6, which are induced by a SARS-CoV-2 virus [75,76]. These secreted molecules further reduce the endothelial junctional proteins, VE Cadherin and zonula occludens-1 (ZO-1) [76], causing increased vascular permeability which mainly occurs due to functional loss of endothelial glycocalyx [77] (Figure 1A). 

#### 3.1.2. Endothelial Bradykinin 1 Receptor (B1R) Trigger during SARS-CoV-2 Infection 

An inflamed vascular microenvironment created during SARS-CoV-2 infection could also lead to the activation of the kallikrein-kinin cascade [78], which exists in two different forms i.e., plasma KKS and tissue KKS [79]. The plasma KKS, including factor XII (FXII), prekallikrein (PK) and high molecular weight kininogen (HMWK), has been strongly associated with vascular inflammation [80] and also reported to activate the intrinsic pathway of blood clotting [81]. Plasma PK circulates in an inactive form and is complexed with HMWK. These PK complexed HMWK binds to its three known endothelial cell binding receptors: urokinase plasminogen activator receptor (uPAR), globular C1q receptor (gC1qR,) and cytokeratin 1 (CK1) [82]. Endothelial bound PK results in the production of its active form, kallikrein [83]. Plasma serine protease kallikrein further catalyzes the liberation of bradykinin, a potent inflammatory substance, from an HMWK [84]. The produced bradykinin either directly exerts inflammatory or vasoactive action by binding to endothelial expressed bradykinin receptor 2 (B2R) or processed by carboxypeptidase N to generate DR9-bradykinin (des-Arg-9-bradykinin) that activates bradykinin receptor 1 (B1R) to deliver inflammatory signals [85]. Both RAS and KKS systems are well appreciated for their indispensable attributes in vascular biology by counter-regulating each other [86], where ACE2 is involved in the degradation of DR9-bradykinin into inactive peptides, while ACE inactivates bradykinin [87] to culminate KKS cascades [85]. When the invaded SARS-CoV-2 virus binds directly to ACE2 receptor, there is an uninterrupted interaction between DR9-bradykinin, and endothelial expressed B1R receptor that promotes the endothelial expressions of (i) pro-inflammatory cytokines and chemokine, such as IL-6 and IL-8 and MCP-1 respectively, and (ii) adhesion molecules, such as ICAM-1 and VCAM, contributing to leukocyte adherence on endothelial cells and (iii) VE-cadherin destabilization [88]. Concurrently, B1R stimulation decreases the (i) expression of vascular endothelial growth factor (VEGF) that is needed to maintain the vasculature and (ii) junctional protein occluden, which is crucial for the tight endothelial junction formation [89]. These effects culminate in vascular hyperpermeability and hence the attributes of non-degraded bradykinins causing vascular hyperpermeability promote blood vessel leakage and angioedema, during SARS-CoV-2 infection, [88], leading to amplified inflammation and SARS-CoV-2 dissemination (Figure 1B).

### 3.2. Endothelial Elemental Changes and Clot Formation during SARS-CoV-2 Infection 

**Figure 2:**
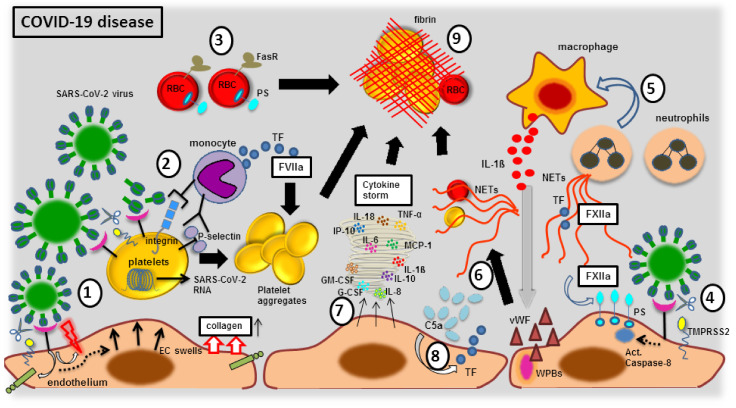
Potential molecular mechanisms involved in coagulopathy during SARS-CoV-2 infection. (**1**) When SARS-CoV-2 binds to ACE2 receptors expressed by endothelial cells (ECs), they damage the endothelial cell membrane, which leads to intracellular changes such as endothelium swelling, collagen exposure, and decreased junctional proteins. The collagen causes platelet aggregation and clot formation. (**2**) Since platelet expresses ACE2, SARS-CoV-2 binds to platelets and activates, as evidenced by the expression of activation and adhesion molecules, P-selectin and integrin, respectively. These adhesion molecules bind to circulating monocytes, which secretes tissue factor (TF) that is known to activate the extrinsic coagulation pathway and leads to clot formation. (**3**) The red blood cells (RBCs) that are recruited to the inflamed vasculature undergo changes in their symmetry and thereby expressed phosphatidylserine (PS), which might lead to thrombin formation. (**4**) SARS-CoV-2 induces caspase 8 in the endothelial cells, which expose PS on the cell surface. This attracts the FXIIa coagulation component to initiate clot formation. (**5**) The recruited neutrophil at the site of inflammation releases neutrophil extracellular traps (NETs) that contain TF and FXIIa. These NETs induce vessel resident macrophages to release IL-1β pro-inflammatory cytokine that acts on vascular endothelium to liberate von Willebrand factor (vWF). Weibel-Palade bodies (WPBs) are elongated secretory organelles specific to endothelial cells that contain von Willebrand factor (vWF) and a variety of other factors. (**6**) These vWFs further induce NETs that serve as the docking site for platelets and RBCs. (**7**) These changes in the vascular microenvironment induce a massive amount of cytokine cocktail called “cytokine storm,” which ultimately induces a coagulation pathway to form a clot. (**8**) Further, complement C5b act on endothelium to induce TF, which leads to clot formation. (**9**) Cumulatively, the molecular and cellular mechanisms that are coordinately responsible for inducing and reinforcing the fibrin clot in COVID-19 positive patients.

One of the major attributes of the SARS-CoV-2 virus is the induction of clot formation within a blood vessel, which draws a special interest to all clinicians and researchers. The incidence of vessel-associated thromboembolism in COVID-19 patients treated in intensive care units (ICU) ranges between 20–50% [90,91,92], but is lowered to 12% (in approximate) by treating with the anticoagulant substances, such as heparin [93]. Despite anticoagulation therapy, a significant proportion of SARS-CoV-2 infected patients still suffer from pulmonary embolism [94]. It is evident that point-of-care thermoelectrometry analyses of blood from COVID-19 patients have shown a rapid clot formation that was linked to increased fibrin polymerization due to the infectious disease [95,96]. Thus, patients with SARS-CoV-2 infection suffer from a severe coagulopathy, which is thought to be one of the main drivers of the pulmonary and extra pulmonary dysfunctions [97]. This coagulopathy is barely linked to routine clinically detectable diagnostic coagulation markers, where the key parameters are elevated D-dimers and fibrin or fibrinogen degradation products [95,96,98,99,100]. While this accounts for disseminated intravascular coagulopathy (DIC) that is commonly noticed among the patients with severe sepsis or acute respiratory distress syndrome, the SARS-CoV-2 induced coagulopathy is thought to be slightly different from DIC, because the prothrombin time (PT), activated partial thromboplastin time (aPTT), fibrinogen levels and platelets count are much less affected [101]. Recently, a three-stage concept of the SARS-CoV-2 induced coagulopathy was proposed: (1) D-Dimer elevation; (2) D-Dimer elevation + PT and aPTT prolongation along with thrombocytopenia; (3) critical illness and laboratory presentation that is more comparable to DIC [102]. Though the underlying molecular mechanisms are still obscure, intensive investigations are being conducted linking coagulopathy to COVID-19 disease, where the cornerstones of the clotting dysregulation during SARS-CoV-2 infection are found to be majorly due to the following crosstalk with the host components, (a) pro-coagulant factors (FXIIa); (b) platelet dysregulation; (c) complement activation; (d) endothelial dysfunction; (e) cellular immune responses; (f) cytokine storm. A scheme illustrating the possible molecular mechanisms and cellular components involved in coagulopathy during the course of COVID-19 disease is shown in Figure 2.

#### 3.2.1. The Blood Vessel Clot Formation with FXIIa Factor 

In comparison to data gathered from several viral infection models [103], it has been predicted that an auto-activation of coagulation factor XIIa (FXIIa) occurs in contact with the SARS-CoV-2 virus [104]. The FXIIa is one of the main proteins of the contact system (CS), which is an innate inflammatory defense mechanism orchestrated against any negatively charged foreign or misfolded proteins, or nucleic acids such as DNA or RNA or microorganisms such as viruses, found in the intravascular niche [105,106]. This speculates that either the whole coronaviruses, which contains conserved negatively charged amino acids located within domain III in the carboxyl end of nucleocapsid proteins [107] or the SARS-CoV-2 RNA that are circulating in the bloodstream [108] are readily capable of inducing FXII-associated intrinsic coagulation pathway to form a pathological thrombus [104]. In fact, a pulmonary vessel wall isolated from COVID-19 positive lung tissues exhibited increased expression and activity of FXIIa [109], showing the commencement of the pathogenic pro-coagulant chain. This FXIIa was activated by an accumulated neutrophil extracellular traps (NETs) indicating an impaired clearance of NETs [109]. Further, the coagulation cascade activation is evidenced by increased plasma levels of thrombin-antithrombin-complexes (TAT) and fibrin among COVID-19 patients [110,111], suggesting an activation by FXIIa [112]. 

#### 3.2.2. The Blood Vessel Clot Formation with Platelet Activation 

In addition to FXIIa, activated platelets exert an intrinsic coagulation pathway that is strongly evidenced during SARS-CoV-2 infection, as COVID-19 patients exhibit thrombocytopenia that is associated with critically severe condition [102,113]. Almost one trillion of platelets circulate in the blood vessel to maintain the vasculature integrity and forms a hemostatic plug during trauma to cease the bleeding [114]. Upon activation, these platelets tend to aggregate to promote clotting and generation of fibrin thrombi and are therefore majorly involved in thrombosis [115,116]. The SARS-CoV-2 virus has the potential to activate platelets, as they bind directly to ACE2 to enter into human platelets and are further primed by TMPRSS2 which is also expressed on platelets [117]. These upregulated ACE2 and TMPRSS2 were confirmed at both RNA and protein levels and were reported to be more comparable with the quantity that was found on human PBMCs [117]. Upon SARS-CoV-2 binding, the ACE2 levels on platelets are readily decreased due to internalization and subsequent degradation [117]. The concentration of SARS-CoV-2 virus with 1 × 10^5^ PFU can induce platelet aggregation within 30 min in the presence of agonists, such as thrombin, collagen or ADP, as evidenced by (i) increased dense granule secretion (ATP release); (ii) integrin α_IIb_/β_3_ activation; (iii) P-selectin (CD62P) expression; (iv) enhanced platelet spreading; (v) accelerated clot retraction [117]. Of note, not only the whole SARS-CoV-2 virus, but also its spike proteins were capable of eliciting platelet activation [117]. These SARS-CoV-2 spike proteins were also shown to enhance (i) platelet aggregation, (ii) P-selectin expression, and (iii) alpha-granule release, by ACE2 phosphorylation leading to downstream signaling with the mitogen-activated protein kinase (MAPK) pathway, including phosphorylation of ERK, P-38 and JNK, and thereby leading to platelet hyperactivity and subsequent release of coagulation proteins, such as factor V and XIII [117]. The SARS-CoV-2 RNA was also detected inside platelets from older COVID-19 patients, irrespective of the disease severity. These disease-sensitized platelets increased the phosphorylation status of PKCδ, which is key regulator platelets activation, granule secretion, and aggregation [118]. Further, activated platelets in patients with COVID-19 have larger platelet volume (MPV), indicating that it is adhered to the endothelial wall to increase in its radius [119]. These large-sized platelets exhibit increased coagulatory potential as they are able to bind more fibrinogen on their surface with a greater level of protein phosphorylation [120,121]. In fact, these subpopulations of large-sized platelets expose phosphatidylserine (PS) to enhance a coating over the platelets by binding to coagulation factors and plasma proteins and providing a pro-coagulant surface and phenotype during the SARS-CoV-2 infection [122]. These platelets also produce extracellular microvesicles containing phosphatidylserine to promote further coagulation [118]. Furthermore, the proinflammatory cytokines IL-1β and IL-6 produced by macrophages and monocytes at inflamed blood vessels [123], have been shown to increase the interaction between platelets and monocytes. Such interactions amplified monocytic expression of tissue factors (TF) to orchestrate extrinsic coagulation pathway, as evidenced by the markers of the hypercoagulatory state, D-Dimers and fibrinogen, among COVID-19 patients [124]. Furthermore, the P-selectin was aberrantly expressed on platelets upon SARS-CoV-2 infection [125,126,127], where the elevated P-selectin is linked to enhanced platelet-leukocyte and platelet-endothelial interactions and activation. Thus, adjoining of platelets with other cells types (leukocytes and endothelial cells) are pro-coagulatory and therefore could be reasoned behind the hypercoagulatory state in COVID-19 disease [128]. 

#### 3.2.3. The Blood Vessel Clot Formation with Complement System Activation

Complement components are the representatives of innate immunity that enhances the attributes of antibodies binding to the invaded pathogens to facilitate their elimination [129]. Complements are mainly generated by the hepatocytes of the liver, however, other cell types such as endothelial cells are also capable of producing the complement components [130,131]. Though complement and coagulation are two distinct systems, there exists an interconnection between the proteins of both the cascades, as evidenced with (i) the activation of complement complex C1 leading to the initiation of the classical complement pathway, by coagulation factor XIIa [132]; (ii) MASP-2 (MBL-associated serine protease 2) is a component of the lectin complement pathway, which significantly contributes to the activation of thrombin and to the formation of fibrin mesh [133]; (iii) the expression of TF on endothelial cells by both the C5a component and MAC (membrane attack complex), when the blood vessels are compromised during SARS-CoV-2 infection [134]. The endothelial TF activity induced by the C5a complement factor further leads to the development of disseminated intravascular coagulation (DIC), as commonly observed among critically ill COVID-19 patients. Of note, plasma levels of C5a complement components were increased among patients with the COVID-19 disease [135]. It is reported that MASP-1 and 2 expression were also increased among these patients, where the N protein of SARS-CoV-2 interacts with MASP-2, causing complement over-activation. The accumulated MASP-2 cleaves prothrombin to thrombin, whereas increased MASP-1 activates endothelial cells through the protease activated receptor PAR-4 to secrete pro-inflammatory cytokines to enhance the chemotaxis of monocytes and neutrophils to the damaged blood vessels. Further, MASP-1 involves in cleaving fibrinogen to fibrin to form a clot [136].

#### 3.2.4. The Blood Vessel Clot Formation with Endothelial Dysfunction

The vascular endothelium is one of the most important players in regulating the coagulatory state to maintain vascular tone and homeostasis, which became evident when endothelial damage led to thrombosis in patient with sepsis [137,138,139]. Likewise, extensive research is being focused on EC dysfunction following SARS-CoV-2 infection. It has been demonstrated that neuropillin-1 positive endothelial cells of small and medium-sized vessels were infected with SARS-CoV-2 [140]. Case reports have shown direct invasion of the SARS-CoV-2 virus into ECs, leading to EC activation and apoptosis as well as pyroptosis [97,141]. Thus, the accumulation of viral elements in the ECs and inflammatory cells cause endotheliitis. Though COVID-19 disease could primarily be regarded as a pneumonia, it could be precisely defined as endotheliitis in multiple organs [142]. Such an inflamed endothelial cell synthesizes and secretes copious amounts of von Willebrand factor (vWF) multimers, which is an acute phase reactant, such as C-reactive protein (CRP) [143]. The vWF are stored in the Weibel–Palade bodies (WPBs) of endothelial cells as a highly prothrombotic protein. Despite heterogeneous function, the vWF multimers are well known for their capability to support platelet aggregation and to safeguard FVIII from proteolytic degradation in blood flow to amplify hemostasis and thromobic events. Of note, patients with COVID-19 disease exhibited elevated levels of vWF [144]. During exocytosis, these vWF are released as very high-molecular-weight (HMW) proteins that exhibit an extraordinarily long platelet catching string such as structure [145]. These HMW vWFs are generally regulated in size by ADAMTS13, a vWF-cleaving protease [146]. However, a significant alteration in the platelet-vWF-ADAMTS13 axis was found among COVID-19 patients, including high vWF levels with high platelet counts and an increase of 3–7 fold of vWF antigen to ADAMTS13 activity ratio that was strongly associated with COVID-19 severity [147]. These data firmly support a sustained ECs activation during SARS-CoV-2 infection [144]. In fact, a comparative analysis from autopsy lungs taken from both COVID-19 and influenza infected patients have shown (i) unique vascular features with severe endothelial injury; (ii) EC infected SARS-CoV-2 virus with disrupted cell membrane; (iii) increased ACE2 positive ECs; (iv) EC swelling, disrupted intercellular junctions and loss of contact with the basal membrane, which were noticed among COVID-19 patients. Of note, COVID-19 damaged pulmonary vessels from a COVID-19 patient have shown disseminated thrombosis with microangiopathy [97]. Thus, endothelial dysfunction remains to be a prime factor for any microvascular damage by switching the vascular equilibrium to vasoconstriction that is assisted with ischemia, inflammation and a pro-coagulant state [148]. Next, the flow of blood, either laminar or turbulent, determines the intensity of shear stress on these underlying vessels associated endothelial cells [149], where laminar shear stress induces NO that makes the vessel wall inflammoresistance. However, turbulent shear stress has been noticed during SARS-CoV-2 infection-causing pathological deformation of vascular wall architecture by inducing vasoconstrictors, platelet aggregation and coagulation factors [150]. Since endothelial glycocalyx maintains the integrity of the vascular structure by regulating shear stress, SARS-COV-2-associated hijacking of the glycocalyx element as its receptor disrupts the endothelial structure and thereby imbalances the shear stress that might be one of the causes for the activation of coagulation factors [151]. 

#### 3.2.5. The Blood Vessel Clot Formation with Cellular Immune Response 

Cellular pathways, including blood circulating and tissue-resident leukocytes, especially monocytes and macrophages, are considered to be the main drivers for the SARS-CoV-2 induced coagulopathy [152]. In COVID-19 patients, an interaction between the platelet and monocyte, which is mediated by P-selectin and integrin α_IIb_/β_3_ expression, has strongly induced the monocytes to express the tissue factor (TF) [124], which is in line with increased TF plasma levels in these patients [153,154]. The secreted TF are well known to induce an extrinsic coagulation cascade [155]. These peripheral blood monocytes arrive to the inflammatory sites and infiltrate the tissue to differentiate into macrophages, which were found on SARS-CoV-2 infected lung tissues [141,156]. Due to the expression of ACE2 receptors, SARS-CoV-2 can easily interact with macrophages, followed by macrophage activation and intensified inflammatory response [157]. Despite antiviral attributes, these viral containing macrophages surprisingly serve to relocate by egressing from the lung tissues to the other organs [158]. The macrophage contained SARS-CoV-2 RNA could trigger TLR7, the RNA sensors, through the adaptor MyD88 and RIG-I through the adaptor MAVS, both of which induce type 1 IFNs production that is activated via the transcription factors STAT1, STAT2 and IRF9, leading to upregulation of caspase-11 and other IFN-inducible proteins [159]. Concomitant activation of caspase-1 and inflammasome pathway leads to macrophage pyroptosis by cleaving gasdermin that forms pores on the plasma membrane to facilitate IL-1β release [160]. Upon inflammasome activation, the pyroptotic macrophage releases TF that further initiates coagulation cascades [160]. The secreted TF evidently plays key role in initiating disseminated intravascular coagulation (DIC), a pathological state of systemic coagulation [161] that was evidently noted among SARS-CoV-2 infected patients [162]. Of note, targeted cell apoptosis remains to be the hallmark during SARS-CoV-2 infection and was shown to have a direct relationship with severity in COVID-19 patients [163]. Human macrophages, T lymphocytes and lung endothelial cells were shown to undergo apoptosis via caspase 8 activation by the SARS virus, where the externalizing phosphatidylserine (PS) from apoptotic cells are shown to possess pro-coagulant properties. Here, the factor XII binds to the apoptotic cells via PS and has shown to be vital for apoptosis-induced thrombin generation [164]. Since macrophages are considered to be the main source of proinflammatory cytokines IL-1β, IL-6, IL-8, IFN-γ and TNF-α, these macrophage-secreted cytokines act on pulmonary vessel ECs, leading to their activation [165]. This has also been shown on COVID-19 lung autopsies, where alveolar macrophages expressing IL-6 and MCP-1 found in the lung tissues, leading to endothelial damage and necrosis that were evidenced by IL-6 secreting macrophages and ECs adhering to vascular thrombi [166]. Apart, neutrophils were also reported to drive the coagulatory cascade activation in COVID-19 patients. Infiltrating neutrophils in the lung tissue of COVID-19 patients have released neutrophil extracellular traps (NETs) upon activation [167]. Elevated NET values were shown in the blood of COVID-19 patients [168], and the released NETs further induce macrophages to release IL-1β, where cooperated action of NETs and IL-1β lead to excessive damage on pulmonary endothelium [169]. This injured endothelium tends to release vWF, which further activates neutrophils as well as blood platelets. As a positive circuit loop, these activated platelets induce inflamed neutrophils to secrete excessive NETs, which serve as a basal structure upon which erythrocytes, fibrins and blood platelets, tend to aggregate and support clot development [169]. In fact, the sera obtained from COVID-19 patients are the strong source for NETs production from control neutrophils [170]. The formed NETs from COVID-19 patients have been shown to carry active TF, which induces the extrinsic coagulation system, and these thrombogenic NETs might also directly induce TF expression on human aortic endothelial cells to enhance pro-coagulant attributes [153]. Additionally, NETs trigger PAI-1 (plasminogen activator inhibitor-1) release from ECs [171,172]. PAI-1 is pro-coagulatory, as it functions to inhibit the tissue plasminogen activator (tPA) and urokinase (uPA), both of which are responsible for fibrinolysis of blood clots [173]. Next, red blood cells (RBCs) that comprise 35–45% of the blood volume are directly involved in thrombin generation and platelet activation [174], by expressing the death receptor FasR and loses its membrane asymmetry to expose negatively charged phosphatidylserine (PS) on its cell surface [175]. This subpopulation of PS-positive (PS^+^) RBCs generates thrombin [174]. Additionally, an increased PS^+^ RBCs positively correlates with biomarkers of coagulation activation [176]. Further, when the RBCs are added to platelet-rich plasma, increased thrombin is being produced [177]. It was shown that RBCs exhibited the greatest deposition of platelet aggregates on a collagen surface under low (150 s^–1^) shear stress rates compared with high (1700 s^–1^) in veins [175] and it is evident that SARS-CoV-2 imbalances the shear stress [178]. These attested functions of blood cellular fractions might render the hypercoagulatory state among SARS-CoV-2 infected individuals.

#### 3.2.6. The Blood Vessel Clot Formation with Cytokine Storm

A dysregulated host immune response to the SARS-CoV-2 virus leads to systemic release of cytokines from inflamed cells, termed as “cytokine storm,” which is evident in severely affected COVID-19 patients [179] and also has been linked to clot formation [180]. The cytokine storm is characterized by high serum levels of various cytokine mixtures, such as IL-1β, IL-6, IL-7, IL-8, IL-9, IL-10, IL-18, IL-33, FGF (fibroblast growth factor), G-CSF (granulocyte-colony stimulating Factor), GM-CSF (granulocyte-macrophage colony-stimulating factor), IFN-γ (interferon-γ), CCL-2 (CC-chemokine ligand 2), IP-10 (interferon-gamma induced protein 10), MCP-1, MIP-1β (Macrophage Inflammatory Protein 1β), PDGF (platelet-derived growth factor), TNF-α, and VEGF [181,182]. These mixed cytokine stews are pro-coagulatory in nature and induce multiple pathways associated with fibrin generations, including platelet activation and aggregation, activation of coagulation factors and complement components, renin-angiotensin system and tissue-resident macrophages [183,184]. In particular, proinflammatory cytokines, IL-1ß, IL-6, IL-8 and TNF-α, resulted in platelet hyperactivation with increased clumps, suggesting that these molecules possess profound effect on rumpled fibrin clot with trapped RBCs [185,186]. The IL-6 cytokine was correlated with the symptomology of COVID-19 patients, including pulmonary inflammation and excessive lung damage [187]. Interestingly, the suppressor of cytokine signaling 3 (SOCS3) was decreased in these patients [188], indicating that the negative regulator of IL-6 cytokine secretion is being hampered. In particular, SOCS3 inhibits STAT-3 activation by using gp130 receptor, a part of the receptor complex for IL-6 cytokine family [189,190,191] and the reduced phosphorylation of STAT-3 decreases IL-6 synthesis. The upregulation of SOCS3 is coordinated in the presence of platelet factor 4 (PF4), a chemokine found in abundance inside the platelet α-granules, to attenuate the STAT-3/IL-17A pathway [192] and further these PF4 are involved in the inhibition activation of the thrombin activatable fibrinolysis inhibitor (TAFI) [193], which protects the formed clot from lysis. Of note, mice with dysfunctional endothelial SOCS3 and activated STAT-3 exhibit pro-thrombotic phenotype, as evidenced by the increased expression of the gene encoding Factor III, urokinase receptor and urokinase that further support the formation of DIC [194]. Apart, chemokines such as IP-10 and MCP-1 were increased among critically ill COVD-19 patients, which were positively correlated with the fibrin degradation products (FDP) and D-dimers [195]. Further, when SARS-CoV-2 infects monocytes probably at the endothelium–monocytes interface, it activates the inflammasome pathway via NLRP3 components and induces pyroptosis to activate caspase 1, which cleaves gasdermin to form pores at the monocyte surface to expel copious amounts of IL-1ß, leading to a hyperinflammatory state [196]. In fact, these key cytokines, including TNF-α, increase the local concentration of thrombin via heterogenous TFs, where thrombin acts on macrophages and platelets, recruited to the endothelial injured site, and cleaves p33 IL-1α which is activated and released by the action of thrombin [197]. These thrombi cleaved IL-1α were found to be elevated among COVID-19 patients, exhibiting severe lung injury [198]. Next, TNF-α plays a key role in impairing an anticoagulant function, by diminishing thrombomodulin activity in the endothelial cells [199]. One such well-known anticoagulant pathway is the protein C-protein S-thrombomodulin pathway, where thrombin–thrombomodulin complex on the endothelial cell surface activates protein C that augments by binding to the endothelial cell protein C receptor (EPCR) and thereby protein C plays a vital role in inactivating the coagulation factors Va and VIIIa, assisted by a cofactor protein S [200]. The cytokine TNF-α downregulates EPCR on the endothelial cells [201], which ceases the further activation of protein C, thereby imbalancing coagulation–anticoagulation system. Taken together, these cytokine storms mediated damage to vascular endothelium activates pro-coagulatory cascade, by expressing TF and vWF molecules and exposing the collagen to blood that breaches the endothelial barrier and enhances platelet binding to ECs to induce platelet activation and aggregation. Concomitantly, a diminished generation of components of anticoagulation and fibrinolysis further boost lung microthrombosis. Such an imbalanced coagulation system generated by copious amounts of cytokines forms DIC and triggers a decompensated thrombocytopenia via excessive aggregation and passive exhaustion of platelets among SARS-CoV-2 infected patients [202]. The list of proinflammatory cytokines with steady state function and association with SARS-CoV-2 related hypercoagulopathy is summarized in Table 1.

## 4. Comprehensive Clinical Observations on SARS-CoV-2 Infected Patients Associating with Cardiovascular Complications

### 4.1. The Impact of COVID-19 on Patients with Cardiovascular Diseases 

The severity of COVID-19 disease is associated with an increased risk, especially, in patients with pre-existing cardiovascular disease. Early reports have indicated that cardiovascular complications are a common comorbid condition observed among SARS-CoV-2 infected patients [224]. When patients with mild or moderate disease were compared with patients with critical illness, cardiovascular diseases, specifically hypertension, diabetes, coronary artery disease and heart failure, were associated with worse prognosis [99,224]. This prompted the health authorities to declare all persons with a history of cardiovascular disease as being at high risk for COVID-19, both in terms of susceptibility and worse disease outcomes. Nevertheless, the data remain controversial regarding susceptibility to SARS-CoV-2 infection. In a meta-analysis of seven early studies from China with 1576 infected patients, the prevalence of hypertension was 21.1%, diabetes 9.7% and cardiovascular disease 8.4% were reported [225], while another systematic review analyzing data from six studies among 1527 COVID-19 patients in China showed that the proportion of patients with hypertension, cardio-cerebrovascular disease and diabetes mellitus was 17.1%, 16.4% and 9.7%, respectively [226]. Although the prevalence of diabetes mellitus and arterial hypertension in the COVID-19 cohort was comparable to that in the Chinese population as a whole, the prevalence of cardio-cerebrovascular disease was significantly higher. However, other data do not support the notion of increased susceptibility of patients with hypertension and/or diabetes to COVID-19. For instance, one study showed that coronary heart disease, hypertension and diabetes mellitus were associated with an increased risk of death in a univariate comparison, but not in a multivariate analysis [227]. These discrepancies in availing clinical data pinpoint that CAD, hypertension and diabetes mellitus, alone do not appear to aggravate the likelihood for an increased susceptibility to COVID-19. Instead several other existing basic and clinical parameters may play some key role in disease progression, which includes age, gender, magnitude of inflammation and pro-thrombotic state, that are the best predictors of multiorgan failure, while pre-existing cardiovascular morbidities should be considered as modulating (risk) factors [228]. Further, the viral defense host cellular components are impaired in these patients with pre-existing cardiovascular disease [229] and with COVID-19 disease [230], where the antiviral effector immune cells, such as mucosal-associated invariant T cells (MAIT cells), were significantly reduced. These patients exhibit decreased levels of natural killer cells (NK cells) [231] and CD8^+^ T cells that are attested by the expressed exhaustion markers such as programmed death-1 (PD-1) and T-cell immunoglobulin and mucin-domain containing-3 (TIM-3) on its cell surface, implicating that the circulating so called antiviral immune cells remain defenseless and are correlated with the COVID-19 disease severity [232]. 

### 4.2. The Impact of COVID-19 on Patients without Cardiovascular Diseases 

Though COVID-19 is primarily a pulmonary disease, it widely affects the cardiovascular system in multiple ways. Since the time when COVID-19 was declared to be a global pandemic by the WHO and in compliance with WHO recommendation of movement restrictions, many countries have strictly imposed a compulsory self-quarantine and restricted movements of their citizenries (lockdown/sit at home/home-office), including compulsion of wearing face masks and to maintain social distancing to prevent the SARS-CoV-2 acquisition or transmission. On the other hand, social detachment or deprivation can significantly contribute to the physical inactivity. In particular, the restriction of physical activities has led to behavioral patterns that can lead to cardiovascular diseases (CVDs), which are the leading cause of the global mortality rate. The increase in this COVID restricted lifestyle [233] and/or substance misuse during the COVID-19 pandemic lockdown as a result of personal limitations in the COVID-19 lockdown is associated with the risk of death from chronic diseases such as cardiovascular diseases [234]. Given the fact that cardiovascular disease is the major comorbidity in COVID-19 patients and is closely related to the severity of COVID-19, during SARS-CoV-2 infection, the cardiovascular system is either directly (especially viral toxicity with sarcomere disassembly) and/or indirectly (e.g., cytokine storm) being affected by unknown pathomechanisms, leading to an increased incidence of cardiovascular associated changes in COVID-19 patients (Table 2) [235,236], where otherwise, these patients remained healthy prior to SARS-CoV-2 infection [27]. Though direct and indirect myocardial injury of COVID-19 and the impact on cardiovascular homeostasis remains elusive, regardless of morbidity, it is at least known, that cardiac injury is associated with higher mortality, due to (ii) admission to the intensive care unit (ICU), (ii) mechanical ventilation and (iii) coagulopathy in patients with COVID-19 [237]. A recent systematic review and meta-analysis could show that, the proportions of cardiac injury were 22%, 28% among hospitalized patients with COVID-19 or severe COVID-19 patients, respectively [238]. The incidences of cardiac injury in patients with COVID-19 >60 years (30%) was about twofold than patients <60 years (15%). Severe cases (42%) have a sevenfold of prevalence cardiac injury than in their non-severe counterparts (6%). Furthermore, cardiac injury is associated with an increased risk of all-cause mortality in patients with COVID-19 (OR 10.11, 95% CI 4.49–22.77) and is vice versa, i.e., in patients with severe COVID-19, cardiac injury is associated with an increased risk of all-cause mortality (OR 16.79, 95% CI: 5.52–51.02) [238]. Recent experimental results have shown that intervention in pathophysiology could prevent damage to the heart. Treatment with the commercially available bromodomain 2-selective bromodomain and extraterminal family (BET) inhibitor apabetalone has shown to reduce the (i) cytokine storm-induced diastolic dysfunction, (ii) ACE2 levels; and (iii) viral infection in cardiomyocytes [239].

**Figure 3:**
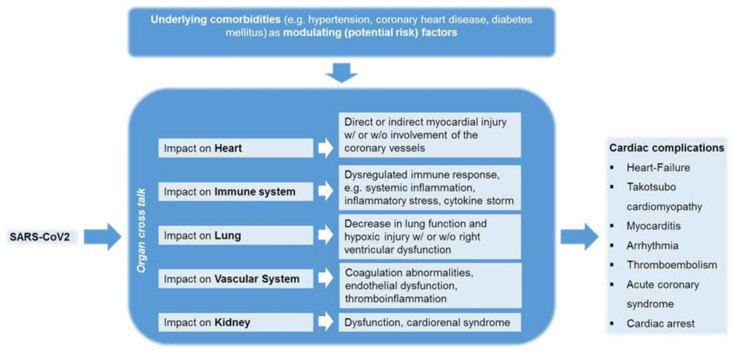
A crosstalk between the organs in inducing cardiac complications that are primarily triggered by SARS-CoV-2 virus among patients with underlying chronic conditions.

COVID-19 has been associated with the development of several cardiac complications, including myocarditis, venous thromboembolic disease, acute coronary syndrome, acute myocardial infarction, arrhythmias, heart failure, cardiomyopathy, cardiogenic shock and cardiac arrest, including and coagulation abnormalities (Figure 3) [240,241]. These cardiac dysfunctional observations were also evident with two previous historical epidemics with coronavirus outbreaks, including SARS and MERS [242]. A Swedish study characterizes the causes of cardiac arrest before and within the COVID-19 pandemic [243]. During the pandemic phase, COVID-19 was involved in at least 10% of all out-of-hospital cardiac arrests (OHCA) and 16% of in-of-hospital cardiac arrests (IHCA). Among COVID-19 cases, very alarmingly, 30-day mortality was increased 3.4-fold in OHCA and 2.3-fold in IHCA [243]. During COVID-19 pandemic, substantial numbers of children exhibited multiorgan inflammatory syndrome in children (MIS-C) or Kawasaki such as disease involving cardiogenic shock, signs of myocarditis, acute left-ventricular dysfunction as a potential risk for a life-threatening condition [244,245]. Though pathophysiology of cardiovascular manifestations is multifactorial and not yet understood in detail in association with COVID-19 disease, several laboratory findings in COVID-19 patients show an increase in high-sensitivity cardiac troponin I (hs-cTnI), creatine kinase (CK)-myocardial band [31] and N-terminal probrain natriuretic peptide (NT-proBNP), which can be interpreted as an expression of myocardial involvement [246]. Whether late cardiac consequences are to be expected in COVID-19 patients was shown in the most recent work by Kotecha et al. [247]. One hundred and forty-eight severely ill COVID-19 patients with troponin elevation, a marker for cardiac damage, were examined by cardio-MRI at a median of 68 days after recovery. More than half (54%) showed abnormalities in the heart. One was myocarditis-like injury (26%) and the other was infarction and/or ischemia (22%) and both pathologies (6%). The results of the above-mentioned studies suggest that several mechanisms associated with SARS-CoV-2 infection may affect the heart, including myocarditis and myocardial ischemia. Major possibilities would be that SARS-CoV-2 is probably triggering these clinical conditions either by eliciting systemic immune responses causing damage to the vital organs, where especially the SARS-CoV-2 virus possesses a high likelihood for inducing cross-reactivity with the host autoantigens as known by the concept called “molecular mimicry” [248] or by causing tissue damage through direct viral invasion and pathogenesis. Furthermore, it can also be postulated that COVID-associated myocarditis could be triggered, especially in individuals who are genetically predisposed to autoimmune diseases [249]. Moreover, vaccine associated adverse cardiovascular reactions are described in the literature, albeit rarely. Although the causality between vaccination and myocardial disease cannot be conclusively determined, there are cases of myocarditis associated with vaccine administration. Analyses of Vaccine Safety Surveillance System (VAERS) data in the USA from 1990 to 2018 in 620,195 individuals have shown that 708 (0.1%) individuals meet the classic definition of myocarditis following vaccination. Similar to the aforementioned case, 72% of those affected described the onset of symptoms ≤ two weeks post vaccination [250]. Systemic events that were correlated to the presented case have been reported more frequently in younger vaccine recipients (16 to 55 years) in contrast to older vaccine recipients (over 55 years) and more frequently after the second dose. Studies have shown that adverse events after vaccination, namely with vaccines against smallpox (59%), Hemophilus influenza type b (22%) and hepatitis B (18%), predominantly cause cardiovascular symptoms. Acute coronary syndromes, ventricular arrhythmias and cardiac arrest have been described [251]. In summary, the incidence of serious adverse events was rare and similar in the vaccine and placebo groups (0.6% and 0.5%, respectively). However, the triggering of cardiovascular complications among COVID-19 vaccine recipients is still obscure and is under intensified investigation. Given the fact that cardiovascular complications could be developed succeeding SARS-CoV-2 infection, the medical authorities must emphasize an urgent need to monitor the cardiology events in surviving COVID-19 patients as well as in COVID-19 vaccinated subjects for the surveillance of cardiac phenotype to prevent the complications in advance.

## 5. Hypothetical Factors Beneath Asymptomatic COVID 19 Patients

Despite patients with distinct symptoms during the course of COVID-19 disease, there are patients who do not exhibit any known signs of the disease and are therefore referred to as asymptomatic. Intriguingly, there are several hypotheses that support the symptomless nature of the disease, which are speculated to be determined by the host factors in terms of susceptibility to infection and the virus-related factors in terms of the degree of virulence. The host associated factors might include (i) insufficient expression of ACE2 receptors on nasopharynx or salivary glands, which could terminate in very feeble infection and could be unnoticed; (ii) polymorphisms in ACE2 gene, causing alteration in affinity binding region and spike protein processing [252]; (iii) polymorphism affecting TMPRSS2 structure and function; (iv) role of sex hormones, as women develop lesser complications than men [253]; (v) type of blood group, as people with A blood group are at higher risk compared to the people with O blood group [254]; (vi) alleles of major histocompatibility complex (MHC) class I, where the human leukocyte antigen (HLA)-B*46:01 tend to load fewer peptides and might increase the susceptibility to infection in comparison to HLA-B*15:03 that enhance T cell immunity [255]; duration of antibodies production [256]. Likewise, the deletion of 15–30 bp in the S1/S2 cleavage site region decreases the virulence of the SARS-CoV-2 virus [257,258]. 

## 6. Different Models to Study the SARS-CoV-2 Virus Interaction with the Cardiovascular System

Since most of the SARS-CoV-2 involved investigation with underlying cardiovascular complications is performed directly on the clinical specimens obtained from COVID-19 patients, a deeper gain in the knowledge for SARS-CoV-2 replication process and disease pathogenesis is required to achieve and design appropriate anti-SARS-CoV-2 targets. To fulfill the needs, suitable model systems still remain to be the major investigative tool, including 2 dimensional (2D) or 3D cell culture systems, organoids, and small or large animals [259]. Due to an increased viremia during the COVID-19 disease course, the blood vessels were presumed to be one of the main targets for the SARS-CoV-2 infection. For this purpose, blood capillary organoids were developed from induced pluripotent stem cells (iPSCs), which resembles native human capillary in terms of (i) CD31^+^ endothelial lining, (ii) PDGFR^+^ pericytes, (iii) existence of lumen and (iv) formation of basal membrane. When these blood vessel organoids are infected with SARS-CoV-2 isolates, increasing viral RNA were detected that were amplified from day 3 to day 6 post-infection, indicating active SARS-CoV-2 virus replication. As expected, supernatants of these infected organoids harbored progeny virus which could efficiently infect the Vero E6 cells, implying the ability to form progeny viruses within capillary organoids. The kinetics of SARS-CoV-2 replication and progeny production were analyzed and subsequently targeted with biologicals such as human recombinant soluble ACE2, which in turn mitigated the SARS-CoV-2 infection [260]. Another study has demonstrated a competent establishment of SARS-CoV-2 infection in 2D human iPSCs-derived cardiomyocytes that expressed ACE2 receptors required for SARS-CoV-2 internalization by iPSC-cardiomyocytes. In addition to ACE2, the establishment of infection was also dependent on cathepsin. Evidence based data show that ACE2 expression is remarkably reduced in cardiomyocytes, upon SARS-CoV-2 infection. These SARS-CoV-2 infected iPSC-cardiomyocytes were subjected to apoptosis and ceased the beating in 72 hours post-infection, demonstrating cardiomyocyte-specific mechanisms induced by a SARS-CoV-2 virus [30]. Besides, this model has also revealed the effectiveness of an anti-viral drug, remdesivir, in curbing the infection. Further, the virus induced cardiotoxicity were also confirmed in a 3D cardiosphere model [31]. Of note, SARS-CoV-2 invasion exhibited cytopathogenic effects on iPSC- cardiomyocytes, inducing cell rounding, formation of grapelike clumps, and complete destruction of cell monolayers together with irreversible pyknotic cell shrinkage, which terminates in myocardial injuries. Furthermore, an array of cytokines and chemokines (IL-6, IL-8, tumor necrosis factor (TNF)-α, CXCL1 and CXCL2) from iPSC-cardiomyocytes were released to assist further injury [261]. Recently employed drugs such as chloroquine (CQ) and azithromycin (AZM) to treat SARS-CoV-2 infection, were also tested on the human embryonic stem cell derived cardiomyocytes to investigate the drug induced cardiotoxicity, which reinforced the findings obtained from clinical data [262]. Apart, a demand for more complex physiological environment could be fulfilled using suitable animal models, including mice, humanized mice, hamsters, ferrets, non-human primates [259]. Since ferrets commonly develop cardiac diseases such as dilated cardiomyopathy, arrhythmias and ferret myocardium has positive force–frequency relationship (FFR) that is quite analogous to human [263], it represents a suitable model to investigate myocardial injury, induced during SARS-CoV-2 infection. Certain strains of Syrian hamster (Bio TO-2) exhibit progressive development of cardiac dysfunctions, including musculocutaneous blood flow associated with the cardiac remodeling that includes atrial hypertrophy and left ventricular dilation, wall thinning, increased collagen density, and decreased mean arterial pressure [264]. Furthermore, non-human primates, including rhesus and cynomologous macaques, are used as models to study the pathogenesis and progression of cardiovascular disease for several years [265]. In fact, ferrets, Syrian hamsters and macaques were shown to exhibit (i) susceptibility to SARS-CoV-2 infection, (ii) disease symptoms; (iii) a predicted binding affinity between SARS-CoV-2 virus and ACE2 receptors [266]. We, therefore, have briefly summarized possible models’ systems that could be used in future to investigate SARS-CoV-2 associated mechanisms in general and also leading to cardiovascular complications.

## 7. Possible Therapeutic Approaches against SARS-CoV-2 Induced Complications 

The availability of a single treatment regimen for mitigating the complications induced by the SARS-CoV-2 virus remains highly unattainable, as COVID-19 disease represents an assemblage of various complications involving vital organs, which have a deleterious impact on the biological system. Therefore, currently, a combination of several drugs is being administered to the patients suffering from COVID-19 disease that is mainly targeted with disease symptoms exhibited by every COVID 19 patient. Since the current review majorly focuses on the complexities involved in the vital organs such as heart and blood vessel, following SARS-CoV-2 infection, we summarize the possible drugs that improve the cardiac injury and vascular-related complications associated with SARS-CoV-2 infection. An anti-viral drug, Remdesivir, is an adenosine analogue that is embedded into the viral RNA chains to terminate in its premature form, resulting in SARS-CoV-2 replication inhibition [267]. It was effective and reduced the death, especially among those patients who were hypoxemic and supported with supplemental oxygen, but not among those with a mechanical ventilator [268]. This direct targeting of the SARS-CoV-2 virus might reduce the viral load and obviously, viral mediated damage to cardiac tissue. However, the management of severe hyper inflammation and hypercytokemia is pivotal to minimize the bystander effects induced by SARS-CoV-2, which accounts for increased cardiovascular complications and mortality in COVID-19 patients. Here, cytokine storm curbing can be achieved by administering humanized monoclonal antibodies against the IL-6 receptor (R), Tocilizumab and Sarilumab, which binds to membrane-bound IL-6R and also to soluble IL-6R. In addition, anti-IL-6 chimeric monoclonal antibodies, Siltuximab, are also given, which directly blocks the IL-6-associated inflammation, and thereby, these drugs improve the clinical outcome by controlling the body temperature together with improved peripheral oxygen saturation and minimized the inflammatory storm [269]. Moreover, there are several ongoing trials for COVID-19 patients to target the macrophage activation to reduce the hyper inflammation state inside the patients, where the drugs are targeted against other pro-inflammatory cytokines and chemokine such as IL-1ß, TNF-α, IFN-γ and CCR5 [270]. COVID-19 patients exhibiting hypoxemia are addressed with the supplemental oxygen support as an initial step during the hospitalization, though the type of support depends on the reported oxygen saturation [271]. Herewith improved hypoxemia of lungs among COVID-19 patients will further minimize the damage to vital organs, such as the heart. Next, COVID-19-associated coagulopathy remains to be a serious complication, leading to venous thromboembolism (VTE), where hospitalized adults with COVID-19 reported incidence of different thrombotic events, ranging between 7.7% and 49%, which is unquestionably higher than the incidence in patients without COVID [272]. The development of thrombosis is considered to be a peculiar feature of the SARS-CoV-2 virus when binds to ACE2, which is expressed on platelets [117]. These hospitalized COVID-19 patients are recommended to take antithrombotic prophylaxis with low-molecular-weight heparin, unless there is a contraindication. Few clinical data have supported the use of fibrinolytic therapy, which might be beneficial in reducing death of COVID-19 patients with acute lung injury [272,273]. These recommendations of drugs for the above-mentioned complications induced by SARS-CoV-2 will certainly minimize the mortality rate [274], with a lesser burden on cardiovascular system, among COVID-19 patients.

## 8. Conclusions

Taken together, the SARS-CoV-2/receptor axis exerts a highly complex pathophysiological mechanism in heart tissues and blood vessel and associated cellular components, indicating SARS-CoV-2 mediated damage in terms of either aggravating the pre-existing cardiovascular conditions or generating a new onset of cardiac dysfunctions in non-chronic and naïve individuals, during the course of infection. Wide tissue expression of the SARS-CoV-2 receptor system or multiple receptor targets to which the SARS-CoV-2 virus binds and facilitates its invasion to establish infection and viral dissemination are considered to be the key determinants for the majority of vital organ dysfunction and failure. This review comprehensively describes several possibly proven and hypothetical molecular mechanisms in cellular fractions of heart and vessel that are likely to occur during SARS-CoV-2 infection, in particular, causing heart failure and injuries to the vascular beds, mediated by host components in terms of vascular leakage and thrombotic events. This review also pinpoints the fact that the SARS-CoV-2/receptor axis plays a pan-pathogenic role in damaging every segment associated with safeguarding and well-functioning of the cardiovascular system. However, until-to-date, several SARS-CoV-2 induced pathomechanisms might either not be noticed properly or could possibly be overlooked due to our knowledge gaps. Since SARS-CoV-2 is a new and highly contagious life-threatening bug that has been unfortunately transmitted to the mankind, existing in the world, would still continue to pose many new unforeseen health challenges to all the affected individuals, which imparts high pressure to all the medical teams and research communities to make continuously strenuous efforts to combat the infection and to gain deeper understandings on the viral-host pathogenesis and to develop new therapeutic interventions, respectively. In order to prevent a complete damage to any vital organs, a thoughtful approach must be conducted through the surveillance and regular monitoring of all surviving individuals via continued follow-ups to prevent post-SARS-CoV-2 associated cardiovascular deteriorations. Further, treatment regimen could include blocking the SARS-CoV-2 receptors at the virus entry level or could minimize or improve vascular injuries by targeting leakage and coagulopathy, at the level of symptomatic disease outcome.

## Figures and Tables

**Table 1:** Cytokine directory in relation to SARS-CoV-2 associated hypercoagulopathy.

Cytokine Name and Type	Primary Functions	Potential Role in SARS-CoV-2 Associated Hypercoagulopathy	References
IL-6, proinflammatory	▪ Induces CRP ▪ Antibody production ▪ Effector T cell development	▪ ↑ Fibrotic clot formation ▪ ↑ Thrombin formation by regulating MMPs and thrombolysis-associated genes ▪ ↑ TF to activate extrinsic coagulation pathway ▪ ↑ Thrombin fragments F1, F2 and thrombin–antithrombin III complexes, indicating coagulation activation ▪ ↑ Fibrinogen and vWF ▪ ↓ Plasma protein S that promote thrombin formation	[203,204,205]
IL-1α, proinflammatory	▪ Promote high grade inflammation ▪ Act as “alarmin”	▪ ↑Monocyte-endothelium interaction that ↑TF activity	[206]
IL-1ß, proinflammatory	▪ Inflammasome activation ▪ Macrophage pyroptosis	▪ ↑Monocyte-endothelium interaction ▪ ↑Macrophage pyroptosis and ↑TF microvesicles to ↑systemic coagulopathy ▪ NLRP3 induced formation of arterial thrombosis	[160,207]
IFN-γ, proinflammatory	▪ Play role in antiviral adaptive immune responses ▪ Activator of macrophages	▪ ↑ Platelet activities ▪ ↑Platelet-monocyte adhesion ▪ ↑TF expression in monocytes ▪ ↑ Thrombus formation ▪ ↓ MMP-9 and ↓VEGF to delay thrombus resolution ▪ ↑ Deep venous thrombosis via ↑NETosis	[205,208,209]
IL-2, growth factor	▪ Promote T cell activation ▪ Promote tolerance by expanding regulatory T cells (Tregs) ▪ Immune suppressive activities of Tregs	▪ ↑ Endothelial activation of PAI-1 ▪ ↓ Fibrinolysis ▪ Impair anti-coagulant function of vascular endothelium	[205,210]
IL-12, differentiation factor	▪ Differentiation of naive to effector cells ▪ Amplify cytotoxic activities of NK cells and CD8+T cells ▪ Role in early inflammation	▪ ↑RBC adhesion with each other and ↑Agglutination causing aberrant hemorheology affecting blood flow ▪ Platelet activation and spreading with ↑hypercoagulation	[211]
IL-18, proinflammatory	▪ Promote high grade inflammation ▪ Facilitates TH_1_ responses ▪ Inflammasome activation	▪ ↑ Microvesicles containing TF that interact with factor VIIa to form clot ▪ Activate NF-kB and vWF to induce deep venous thrombosis ▪ ↓ Tissue plasminogen activator (tPA)	[212,213]
TNFα, proinflammatory	▪ Promote high grade inflammation ▪ Facilitate tissue pathology	▪ Induction of TF on endothelial cells ▪ ↓ Thrombomodulin activity for anticoagulant function ▪ ↓ EPCR expression on endothelial cells and ↓ Protein C activation needed for anticoagulation ▪ ↑ Release of PAI-1	[201,214,215,216]
GM-CSF, hematopoiesis cytokine, growth factor	▪ Induces stem cells to generate subpopulation of granulocytes and monocytes ▪ Involved in myelopoiesis	▪ ↑ Monocytic production of TF ▪ Activation of coagulation system	[217,218]
MCP-1, proinflammatory	▪ Chemoattractant factor for monocyte–macrophages system	▪ ↑ D-dimers ▪ Accumulates TF activities	[219]
IL-10, anti-inflammatory	▪ Regulation of the immune responses ▪ Expansion of Tregs	▪ No direct evidence for thrombus formation ▪ Few reports support on cytokine activation by recombinant (r) IL-10	[220,221,222]
IL-17A, proinflammatory	▪ Role in allergic responses ▪ Induce inflammation against extracellular pathogens	▪ Recombinant (r) IL-17A promotes deep vein thrombosis ▪ ↑ platelet activation and aggregation; neutrophil infiltration and endothelium activation	[223]

Where, IL—interleukin; CRP—C-reactive protein; MMPs—matrix metalloproteinase; TF—tissue factor; NLRP—NLR family pyrin domain containing 3; IFN—γ –interferon gamma; VEGF—vascular endothelial growth factor; NETosis—Neutrophil extracellular traps; PAI—1-plasminogen activator inhibitor-1; RBC—red blood cells; NK—natural killer; vWF—von Willebrand factor; TH_1_—T helper 1; NF-kB—nuclear factor kappa-light-chain-enhancer of activated B cells; TNFα—tumor necrosis factor alpha; EPCR—endothelial protein C receptor; GM-CSF—granulocyte monocyte colony-stimulating factor; MCP—1-monocyte chemoattractant protein-1.

**Table 2:** Potential direct and indirect mechanisms of SARS-CoV-2-mediated myocardial injury (modified according to Kurz et al. [228]).

ACE2-Mediated Direct Damage	Hemodynamic Strain of Right Ventricle	Hypoxia-Induced Myocardial Injury	Cardiac Vascular Damage	Systemic Inflammatory Response Syndrome
▪ Increased affinity to ACE2 ▪ Myocarditis-like injury ▪ RAAS dysregulation	▪ Pressure/volume increase in RV ▪ Increased RV wall stress ▪ RV dysfunction	▪ Energy depletion ▪ Intracellular acidosis ▪ Mitochondrial damage	▪ Endotheliitis ▪ Thrombus formation ▪ Lung embolism	▪ Cytokine storm ▪ Dysregulated immunocyte ▪ Uncontrolled inflammation

Where, ACE2- angiotensin converting enzyme 2; RAAS- renin angiotensin aldosterone system; RV-right ventricular.

## Abbreviations

COVID-19	Coronavirus Disease-2019
SARS-CoV-2	Severe Acute Respiratory Syndr
WHO	World Health Organization
ACE2	Angiotensin Converting Enzymes-2
TMPRSS2	Transmembrane Protease, Serine 2
ECs	Endothelial Cells
ROS	Reactive Oxygen Species (ROS)
HIF-1α	Hypoxia Inducible Factor-1 alpha
STAT-1	Signal transducer and activator of
IL	Interleukin
ADAM-17	Action of a Disintegrin and Metallo
TNF-α	Tumor Necrosis Factor-alpha
NOX	NADPH oxidase
PKR	Protein Kinase R
Nrf2	Nuclear Factor E2-related Factor 2
TLR	Toll-like Receptor
ROS	Reactive Oxygen Species
VCAM	Vascular Cell Adhesion Molecules
TACE	Tumor Necrosis Factor-a Converting Enz
RBD	Receptor Binding Domain
TGF-β	Transforming Growth Factor-Beta
RNA	Ribonucleic Acid
RAS	Renin–Angiotensin System
CAD	Coronary Artery Disease
KKS	Kallikrein–Kinin System
B1R and B2R	Bradykinin Receptors
DR9-bradykinin	des-Arg-9-bradykinin
gC1qR	Globular C1q Receptor
Ang II	Angiotensin II
ICAM-1	Intracellular Adhesion Molecules-1
HMWK	High Molecular Weight Kininogen
NO	Nitric Oxide
eNOS	endothelial nitric oxide synthase
vWF	von Willebrand factor
NETs	Neutrophil Extracellular Traps
TF	Tissue Factor
LDL	Low Density Lipoproteins
PAI-1	Plasminogen Activator Inhibitor-1
WPBs	Weibel-Palade bodies
ZO	Zonula Occludens
DIC	Disseminated Intravascular Coagulation
PS	phosphatidylserine
RBCs	Red Blood Cells
EPCR	Endothelial Cell Protein C Receptor
NLRP3	NLRP- NLR family pyrin domain contain
IP-10	Interferon gamma-induced protein 10
CS	contact system
FasR	FS-7-associated surface antigen Recept
iPSCs	Induced Pluripotent Stem Cell
VTE	Venous Thromboembolism
MCP-1	MCP-1-monocyte chemoattractant prote
OHCA	Out-of-Hospital Cardiac Arrest
IHCA	In-of-Hospital Cardiac Arrest
VEGF	Vascular endothelial Growth Factor
IFN-γ	Interferon-gamma
GM-CSF	Granulocyte-Monocyte Colony Stimulating Fac
